# Prescriptions and Dispensing

**Published:** 1909-01-02

**Authors:** 


### Therapeutics.

### PRESCRIPTIONS AND DISPENSING.

### SUPPOSITORIES.

Under the head of suppositories may [illegible]lso be included medicinal pessaries and bougies, urethral or nasal, since all these differ only in size and shape. Suppositories only are officinal.

For these forms of administering medicines the requirements are: (1) the uniform distribution of the active ingredients throughout the mass; (2) sufficiently accurate division into doses; (3) a vehicle which gives sufficient hardness for the suppository to be easily introduced, but which shall liquefy wholly or in great part at body temperature; and (4) the absence of irritating qualities in this vehicle.

The usual shape for suppositories and pessaries is that of a blunt cone with a flat base—projectile form. Recently another shape, tapering at both ends, has come into favour, the advantage of this being that as soon as the widest part of the suppository has passed the sphincter ani the pressure of the latter ensures it being carried well up into the rectum.

Urethral bougies are generally cylindrical, or else cones tapering at first very slightly to near one end, and then more rapidly to a blunt point.

Nasal bougies have a shape intermediate between that of urethral bougies and rectal suppositories. On the manufacturing scale they are made in special moulds of various sizes.

It may easily happen that a suppository is rather urgently needed, and must be made at home. Serviceable moulds may be made as follows: Soften the end of a stick of sealing-wax and model it with the fingers into the shape required; or trim a piece of wood with a pen-knife until it has the requisite tapering sides and rounded point, finally smoothing it well with fine sand-paper. Then make a rather stiff mixture of linseed meal and water, and put it into a box of greater depth than the height of the suppositories to be made; carefully wrap tin foil round the model of sealing wax or wood, creasing it as little as possible; push it down into the linseed mixture and withdraw the stick, leaving the tinfoil surrounded by the firm linseed. As many moulds as may be needed can be prepared, ready to receive the molten mixture.

Bougies may be moulded in a piece of glass tubing. One end of the tubing is dipped into the molten material, and suction applied to a piece of rubber tubing attached to the other end. When the tube is filled to a sufficient height the rubber tubing is pinched, and the glass tube transferred to cold water till the contents have set firmly. T[illegible] cylinders of material may then be pushed out wit[illegible] small glass or other rod that can pass down the gl[illegible]s tube; each bougie can then be cut to size, and if nec[illegible]sary shaped and smoothed with a clean cloth.

Oil of theobroma (cocoa-butter) is the basis employed in mos[illegible] cases. It is used in all the officinal suppositories [illegible]xcept that of glycerin, in which gelatin is employed instead. Oil of theobroma has the great advantage that it is firm at the ordinary temperature, but melts entirely some degrees below body temperature. It is also less prone to become rancid than are most other fats. When the nature of the medicament is such that the melting point of the mixture is lowered to an inconvenient extent, or in very hot weather, or for use in the tropics, some white wax may be added; from one to three grains of wax for each 15-grain suppository does not raise the melting point above that of the body. On the other hand, when the other ingredient or ingredients are such that the melting point of the mixture would be too high, more or less lard may be added in place of part of the cocoa-butter.

Various other materials have been proposed for general use as a basis, the chief being cocoa-nut stearin, and a mixture of stearic and oleic acids. They do not, however, possess any advantage over cocoa-butter, and they have not been adopted to any considerable extent.

Certain medicaments are best with a non-fatty basis; a jelly composed of glycerin and gelatin is then employed. Glycerin itself is often required in the form of a suppository for the relief of constipation, especially in children. It may be made into a stiff jelly with gelatin, as in the officinal formula for suppositoria glycerini, which contains 70 per cent. of glycerin; or a mass may be made by the aid of sodium stearate, as in the United States Pharmacopœia, when as much as 95 per cent. of glycerin can be attained in the suppository.

Glycerin suppositories made with sodium stearate are very hygroscopic, and each should be wrapped separately in tinfoil or waxed paper. This precaution is less necessary for those made with gelatin.

When a jelly is required merely to act as the basis for a medicament, and the local therapeutic effect of the glycerin is not required, it should be made with a much smaller proportion of glycerin than that contained in the officinal suppository. The following is a suitable composition:—

| | |
|---|---|
| Gelatin ... ... ... ... ... | 10 parts. |
| Water ... ... ... ... ... ... | 40 parts. |

Soak for two hours; then dissolve by the aid of gentle heat; and add

| | |
|---|---|
| Glycerin ... ... ... ... | 15 parts. |

The whole should then be evaporated on a water-bath until all the water has been driven off; that is to say, until the whole weighs 25 parts.

Substances soluble in water, if required to be used in quantities that cannot be made into a homogeneous mixture with cocoa-butter, can be dissolved and mixed with the melted gelatin base. The proportions of gelatin and glycerin can be varied according to the purpose for which the mass is required, and in some cases it is best not to evaporate off the whole of the water, thus obtaining a softer mass.

We have already referred to dispensing difficulties which are noted from time to time in the *Pharmaceutical Journal*; they are there brought forward as concrete examples of prescriptions sent by doctors to chemists to be made up. In our next articles upon Prescriptions and Dispensing we propose to give a dozen or more such actual prescriptions, pointing out the difficulties that arise from each.

(*To be concluded.*)

